# Multi-modal imaging of adhesive capsulitis of the shoulder

**DOI:** 10.1007/s13244-016-0491-8

**Published:** 2016-04-23

**Authors:** Marcello Zappia, Francesco Di Pietto, Alberto Aliprandi, Simona Pozza, Paola De Petro, Alessandro Muda, Luca Maria Sconfienza

**Affiliations:** Dipartimento di Medicina e di Scienze della Salute, Università degli Studi del Molise, Via De Sanctis 1, 86100 Campobasso, Italy; Dipartimento di Diagnostica per Immagini, AORN A. Cardarelli, Via Antonio Cardarelli 9, 80131 Napoli, Italy; Servizio di Radiologia, IRCCS Policlinico San Donato, Via Morandi 30, 20097 San Donato Milanese, Milano, Italy; Dipartimento di Radiologia, Azienda Ospedaliera Città della Salute e della Scienza, Centro Traumatologico Ortopedico, Via Zuretti 29, 10126 Torino, Italy; UO Radiologia 1, IRCCS Azienda Ospedaliera Universitaria San Martino-IST, Viale Benedetto XV 10, 16132 Genova, Italy

**Keywords:** Shoulder, Adhesive capsulitis, Ultrasound, Arthrography, Magnetic resonance

## Abstract

**Abstract:**

Adhesive capsulitis of the shoulder is a clinical condition characterized by progressive limitation of active and passive mobility of the glenohumeral joint, generally associated with high levels of pain. Although the diagnosis of adhesive capsulitis is based mainly on clinical examination, different imaging modalities including arthrography, ultrasound, magnetic resonance, and magnetic resonance arthrography may help to confirm the diagnosis, detecting a number of findings such as capsular and coracohumeral ligament thickening, poor capsular distension, extracapsular contrast leakage, and synovial hypertrophy and scar tissue formation at the rotator interval. Ultrasound can also be used to guide intra- and periarticular procedures for treating patients with adhesive capsulitis.

***Key Points*:**

*• Diagnosis of adhesive capsulitis is mainly based on clinical findings.*

*• Imaging may be used to exclude articular or rotator cuff pathology.*

*• Thickening of coracohumeral and inferior glenohumeral ligaments are common findings.*

*• Rotator interval fat pad obliteration has 100 % specificity for adhesive capsulitis.*

*• Ultrasound can be used to guide intra- and periarticular treatments.*

## Introduction

Adhesive capsulitis (AC) of the shoulder is a clinical condition characterized by progressive limitation of active and passive mobility of the glenohumeral joint, generally associated with high levels of pain [1].

Although the diagnosis of AC is based mainly on clinical examination, various imaging modalities, including arthrography, ultrasound, magnetic resonance imaging (MRI), and MR arthrography (MRA), may help to confirm the diagnosis and to detect the presence of associated characteristics such as rotator cuff abnormalities or intra-articular pathology [2].

In this paper, we review the major clinical and imaging findings encountered in patients with AC.

## Epidemiology and pathogenesis

AC was initially described by Duplay in 1872, who called the condition “scapulohumeral periarthritis” In 1934, Codmann used the designation “frozen shoulder” [1], and the term “adhesive capsulitis” was first introduced in 1945 by Neviaser [3].

The prevalence of AC in the general population is 2–5 %, with most patients over 40 years of age and with women slightly more affected than men [4]. Contralateral shoulder involvement is uncommon [4]. Several predisposing factors have been reported, including trauma, hemiplegia, cerebral haemorrhage, hyperthyroidism, cervical discopathy, diabetes, hypercholesterolemia, and inflammatory lipoproteinemia [5].

The pathogenesis and macroscopic abnormalities of AC were first reported in 1945 by Neviaser et al., who described this condition as thickening and contraction of the glenohumeral joint capsule [3]. The authors also noted the adhesion of the capsule to the humeral head, thus introducing the concept of AC. More recent studies have noted abnormalities of the rotator cuff interval, and in particular, the coracohumeral ligament [6]. Bunker et al. found a higher prevalence of cytokines and growth factors in tissue specimens of patients with AC compared to controls, and also reported the absence of metalloproteinase MMP-14, needed to activate the proteolytic enzyme gelatinase A [7]. Some years later, proliferative synovitis was associated with AC, often involving the sheath of the long head of the biceps tendon, and chronic inflammatory involvement of the supraspinatus tendon was also reported. Macnab suggested that autoimmunity might be responsible for the condition as a whole [8]. At any rate, the exact etiology of the condition is still unknown.

Various classifications of AC have been proposed. The most widely used is that of Lundberg et al., who classified the condition as primary when a clear cause could not be established, and secondary when AC capsulitis occurred after a definite event (e.g., trauma). However, other classifications based on degree of capsular retraction, degree of movement, and arthrographic findings have been reported.

## Clinical findings and treatment

The most typical features of AC are pain associated with progressive stiffness and loss of external rotation movements of the shoulder [9]. The loss of other motion may also be present, depending on the area of the capsule most affected. Pain may be reported anteriorly or posteriorly, occasionally extending over the biceps tendon, especially while resting in bed; however, in most cases, pain cannot be localized reliably [4].

Generally, three separate phases can be identified:

- "Freezing" phase, with duration varying from of 10 to 36 weeks. The main symptom is pain, especially during the night, with little response to oral administration of non-steroidal anti-inflammatory drugs. In this phase, the range of motion begins to narrow.
- "Frozen’" phase, with a duration of 4 to 12 months. Pain gradually diminishes while stiffness persists, with an almost complete loss of external rotation movement.
- "Thawing" phase, lasting between 12 and 42 months [10], although some authors have reported stiffness persisting up to 7 years [11]. In this phase, stiffness gradually disappears and range of motion is gradually recovered.

The aim of treatment is to reduce pain and restore the range of motion, and should be tailored to the severity of symptoms and disease duration. Several treatment options have been reported for AC, but the evidence is still poor, whether these options are used alone or in various combinations [12].

Physiotherapy is typically the first therapeutic approach, with the immediate goal of preventing further limitation of movement and then restoring the range of motion [13]. Steroids are commonly used to treat AC, administered both orally and intra-articularly. These are usually accompanied by physiotherapy, and thus discerning the advantages treatment or another is difficult. Some studies have reported a rebound of symptoms at the end of steroid treatment; thus, the pros and cons should be carefully evaluated in every patient [14–16]. Hydrodilation of the glenohumeral joint capsule is another practicable option [17, 18]. A systematic review of this treatment found the procedure to be effective, but there is little evidence of superiority to other treatments [19]. Suprascapular nerve block can be used to reduce pain sensitivity and to improve range of motion, and can be easily performed under ultrasound guidance [16]. Randomized studies [20] have demonstrated pain reduction and improved range of motion in treated patients compared to control groups. Other treatment options are available, including glenohumeral joint mobilization under sedation and arthroscopic or open capsular release [21]. However, all invasive procedures should be reserved for cases that do not resolve spontaneously or respond to conservative therapies.

## Imaging

The diagnosis of AC is usually clinical. Imaging is most helpful in cases with less severe clinical symptoms that might be misdiagnosed as rotator cuff tears, bursitis, or other conditions.

### Plain radiography

In patients with AC, plain films are usually unremarkable. However, plain radiography may be useful for detecting the presence of associated features, such as osteophytes, loose bodies, or periarticular calcifications.

### Conventional arthrography

Conventional arthrography has historically played an important role in the evaluation of patients with AC, having been used for both diagnostic and therapeutic purposes [22]. The process consists in the intra-articular injection of diluted iodinated contrast, after which standard and supplementary shoulder projections are obtained.

**Fig. 1:**
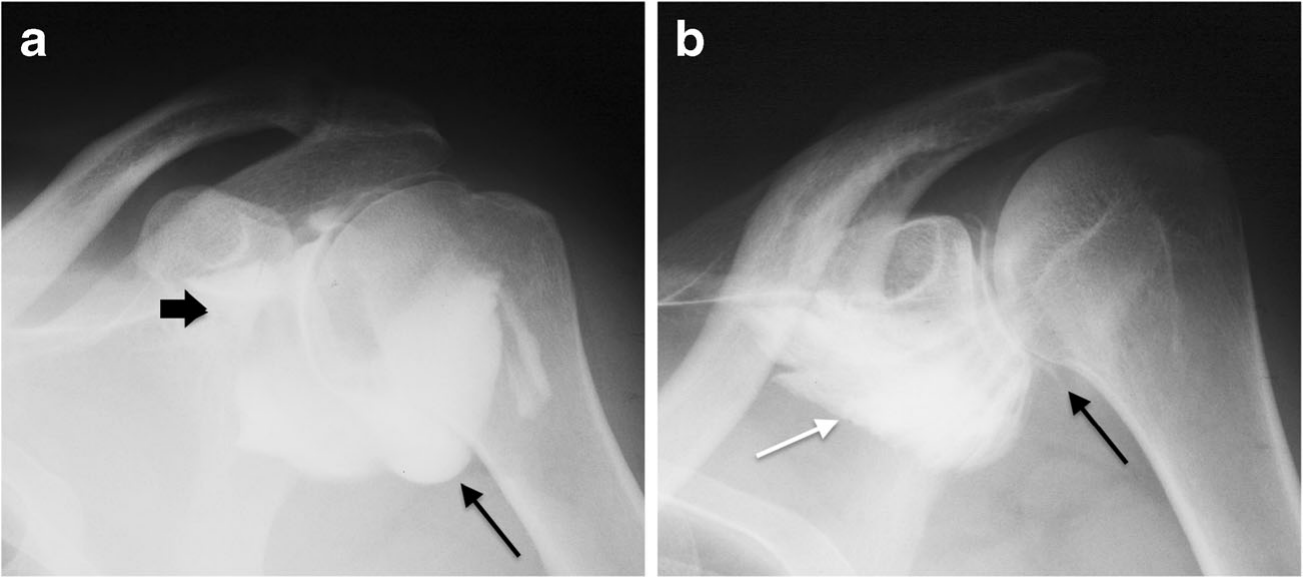
Conventional arthrography, anteroposterior view. (**a**) Normal distension of the axillary recess (*black arrow*) and the subscapular recess (*thick arrow*). (**b**) Reduced distension of the axillary recess (*black arrow*) and subscapular recess associated with medial leakage of contrast (*white arrow*) in a patient with adhesive capsulitis

A number of findings on conventional arthrograms suggest a diagnosis of AC. These include reduced capsular distension with irregular internal profile and internal septa, which is associated with medial leakage of contrast, lack of distension of the subscapular bursa, and atypical contrast leakage in the sheath of the biceps [23] (Fig. 1). Although reduced capsular volume is a common finding, no clear data exist regarding its quantification on conventional arthrography. Harryman et al. reported that joint capacity in patients with AC was lower than 10-12 ml [24].

### Ultrasound

**Fig. 2:**
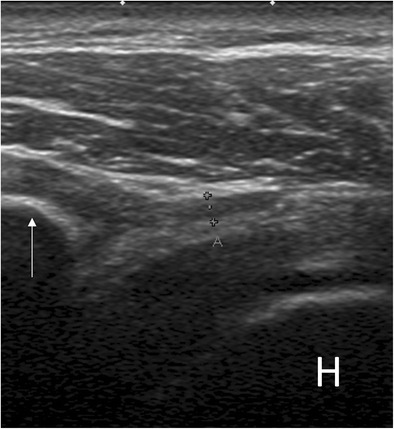
Long-axis ultrasound scan of the proximal portion of the coracohumeral ligament (calipers) in a patient with adhesive capsulitis. The ligament is hypoechoic and thickened (1.8 mm). *H* humerus, *arrow* coracoid process

**Fig. 3:**
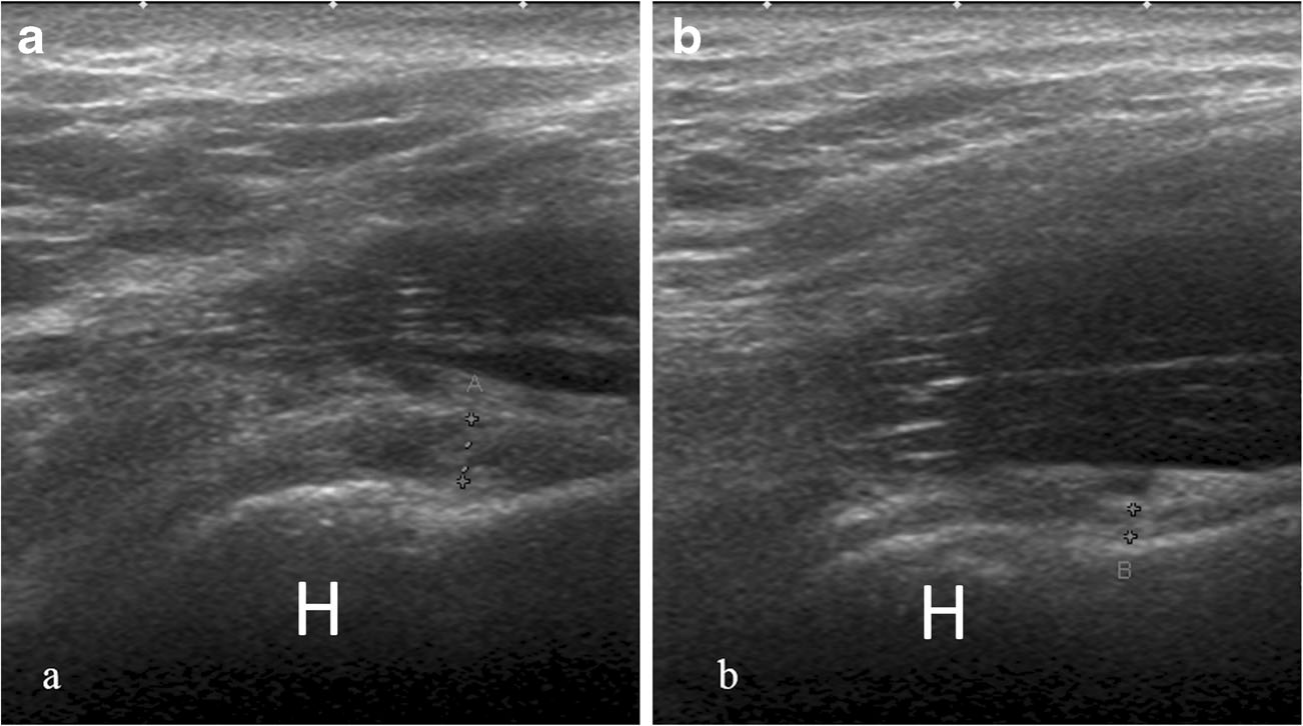
Axillary long-axis view of the inferior glenohumeral ligament with arm in abduction. (**a**) Thickening of the inferior capsular profile (calipers, 3.3 mm) in a shoulder affected by adhesive capsulitis. *H* humerus (**b**) In the contralateral shoulder, the capsule has normal thickness (calipers, 1.5 mm)

**Fig. 4:**
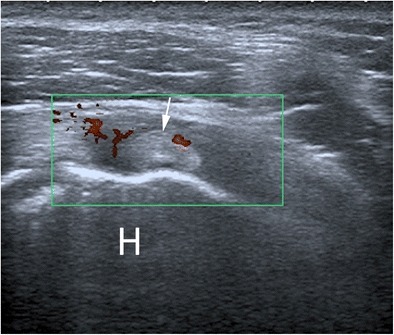
Evaluation of the rotator interval in a patient with adhesive capsulitis for 3 weeks. The power Doppler signal is clearly seen within hypoechoic scar tissue (*asterisks*). *H* humerus, *arrow* biceps tendon

The role of ultrasound in the diagnosis of AC is still controversial. AC was not included among clinical indications for musculoskeletal ultrasound issued by the European Society of Musculoskeletal Radiology in 2012 [25]. However, various studies have illustrated some specific findings that may help to orient the diagnosis of AC. Homsi et al. reported that the coracohumeral ligament was significantly thicker in patients with AC than in asymptomatic volunteers (3 mm vs. 1.34 mm) [26]. However, the time of symptom onset was not taken into account, and thus results were not correlated to the clinical phase of the disease. (Fig. 2). In another paper, Michelin et al. [27] demonstrated that the axillary pouch was thicker in patients with AC than in asymptomatic controls (4 mm vs. 1.3 mm) (Fig. 3). Other ultrasound findings in AC include a hypoechoic appearance of the coracohumeral ligament and the presence of power Doppler signal at the rotator interval (Fig. 4). Doppler signal results from hypervascular hypoechoic scar tissue (proliferation) which develops at the rotator interval and other portions of the capsule in patients with AC. Lee et al. calculated 97 % and 100 % sensitivity and 87 % and 100 % specificity, respectively, of these signs. The power Doppler signal also seems to be present more during the freezing phase than the other phases [28]. Dynamic evaluation may play a role, as rotation of the humeral head is at least partially reduced in patients with AC, thus limiting the gliding and visibility of the supraspinatus tendon under the acromion [29]. Because ultrasound may be difficult to perform in these patients, MRI may be performed first to exclude rotator cuff abnormalities.

### MRI and MRA

**Fig. 5:**
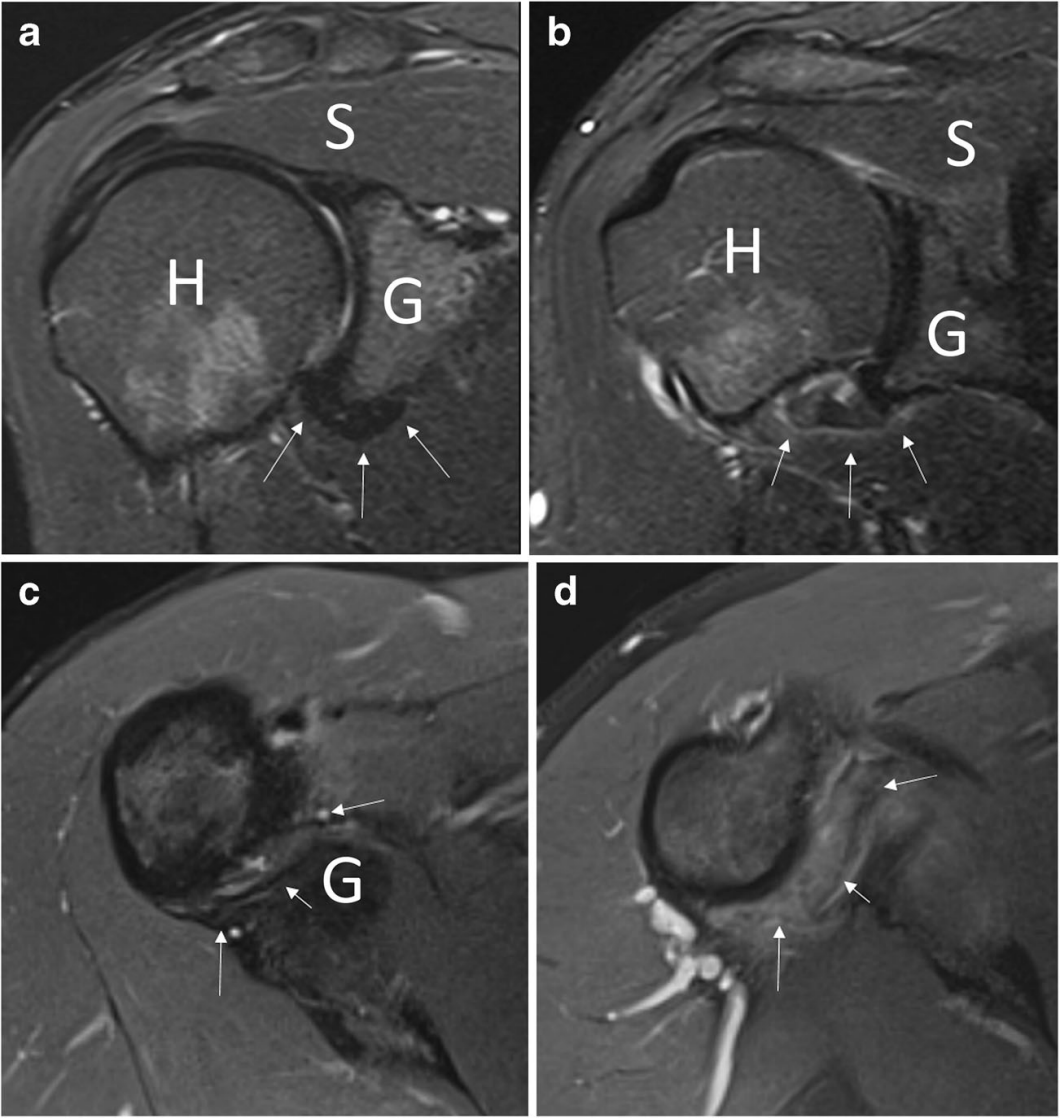
Coronal oblique T2-weighted fat-saturated (**a**, **b**) and axial proton density fat-saturated (**c**, **d**) images. In a healthy subject (**a**, **c**), the capsular recess has normal signal intensity (*arrows*), while in a patient with adhesive capsulitis (**b**, **d**), clear signal hyperintensity can be seen (*arrows*). *H* humerus, *G* glenoid, *S* supraspinatus tendon

**Fig. 6:**
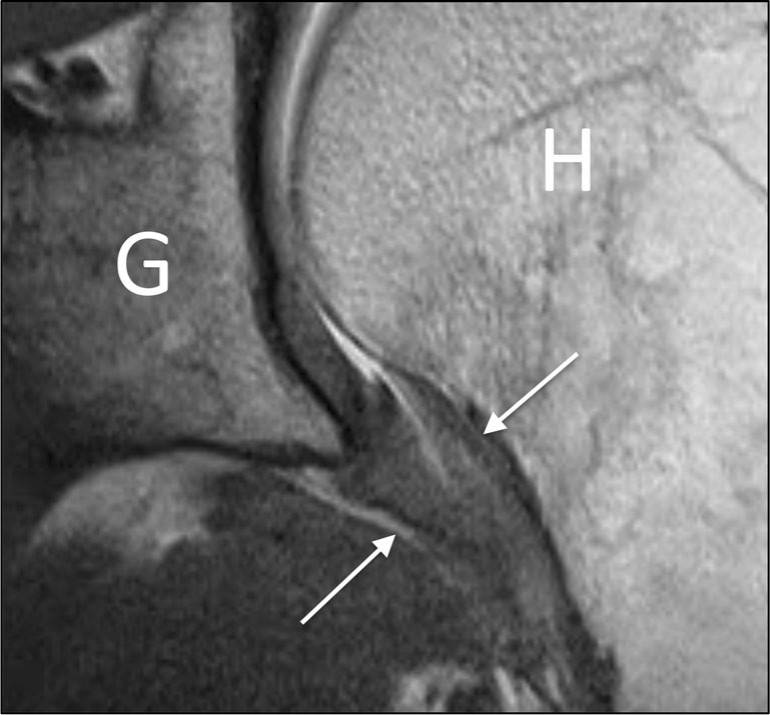
Coronal oblique proton density image in a patient with adhesive capsulitis. The axillary pouch (*arrows*) is thickened. *G* glenoid, *H* humerus

A number of signs of AC on MRI and MRA have been reported. The increased signal intensity of the inferior glenohumeral ligament on fat-saturated T2-weighted sequences was found to have 85.3–88.2 % sensitivity and 88.2 % specificity, with excellent interobserver agreement [30] (Fig. 5). Some authors have proposed that high pericapsular signal intensity corresponds to hypervascular synovitis typically seen in the frozen phase of AC [31]. Intravenous administration of contrast agents (i.e., indirect MRA) may be helpful for identifying enhancement of capsular and synovial structures related to the ongoing inflammation [32, 33]. Furthermore, post-contrast enhancement of the axillary pouch seems to correlate to the reduced range of motion of the shoulder during AC [34]. Researchers have reported no significant differences in diagnostic performance between MRI and indirect MRA in identifying abnormalities of the axillary pouch, and the implication of this structure in AC has long been debated. Emig et al. was the first to report such an association, noting that axillary pouch thickening over 4 mm on MRI demonstrated 65 % sensitivity and 90 % specificity [35]. Subsequent studies, all performed using MRA, showed contrasting results. Manton et al. reported that this sign was not specific to a diagnosis of AC [36]. Lee et al. found a mean capsular thickness of 2.97 mm in patients with AC compared to 1.86 mm in healthy controls [37]. Conversely, Mengiardi et al. found no difference in capsule thickness between patients with AC and controls [2]. Lastly, Jung et al. demonstrated that capsular thickness greater than 3 mm at the axillary recess on coronal oblique non fat-saturated T2-weighted sequences was a specific sign of AC (Fig. 6) [38].

**Fig. 7:**
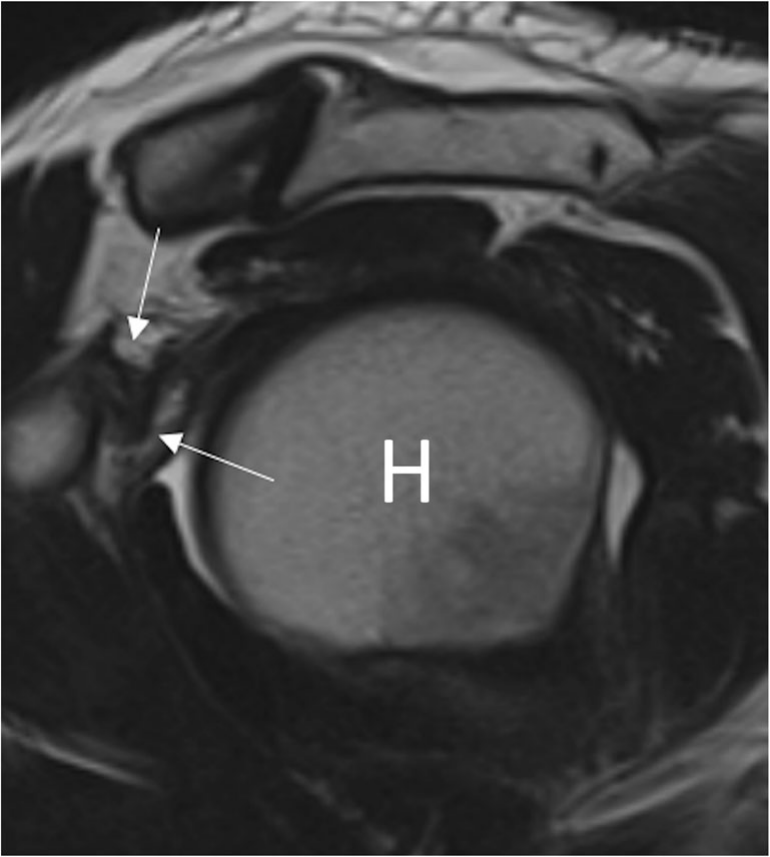
Sagittal oblique T2-weighted image in a patient with adhesive capsulitis. The coracohumeral ligament (*arrows*) is markedly thickened

**Fig. 8:**
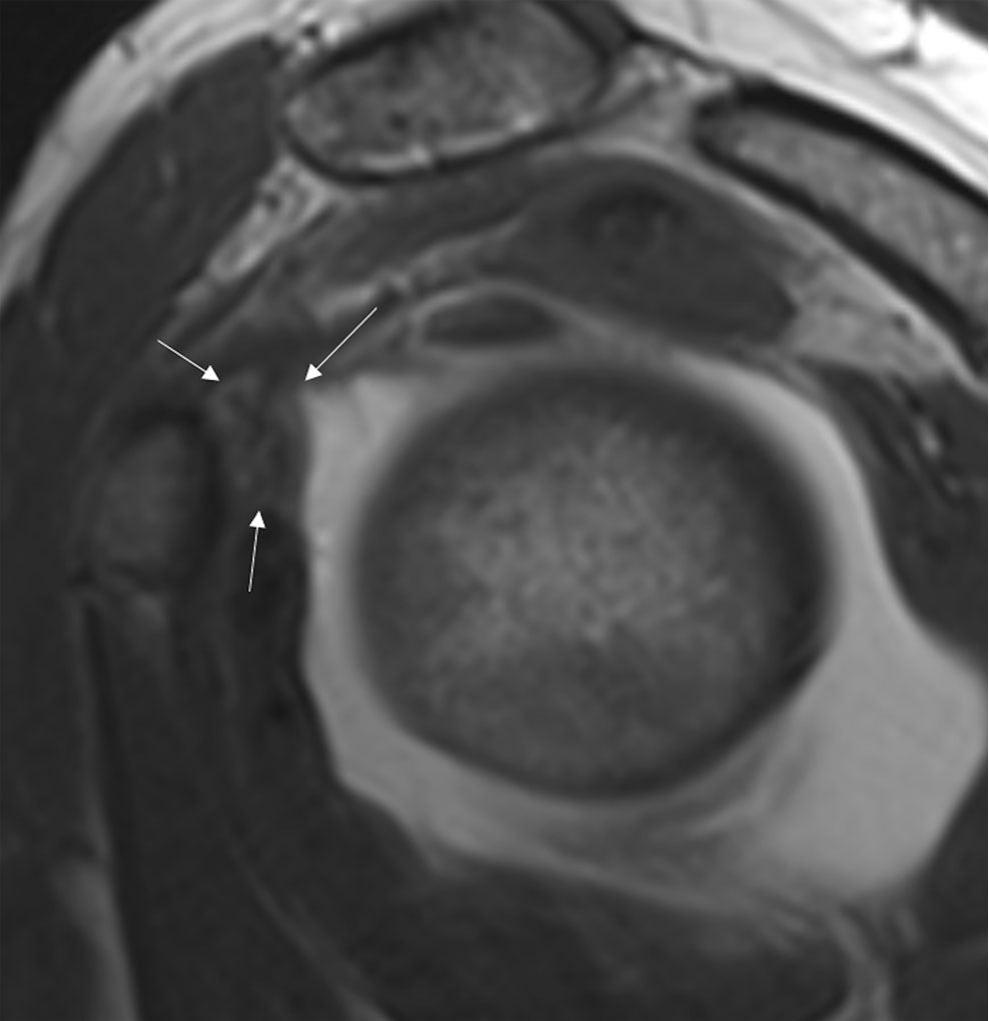
MR arthrography, sagittal oblique T1-weighted image. In a patient with adhesive capsulitis for 15 weeks, the fat triangle (*arrowheads*) signal is considerably reduced

MRA can also be used to detect abnormalities over the rotator cuff interval [39, 40]. Thickening of the coracohumeral ligament and capsule at the rotator cuff interval has high specificity but low sensitivity for the diagnosis of AC [2, 41]: a coracohumeral thicker than 4 mm has 59 % sensitivity and 95 % specificity, while a 7-mm threshold for capsule thickness has 64 % sensitivity and 86 % specificity (Fig. 7) [2]. Mengiardi et al. showed that the obliteration of the triangular fat pad inferior to the coracohumeral ligament had the highest specificity (100 %) but low sensitivity (32 %; Fig. 8) [2].

**Fig. 9:**
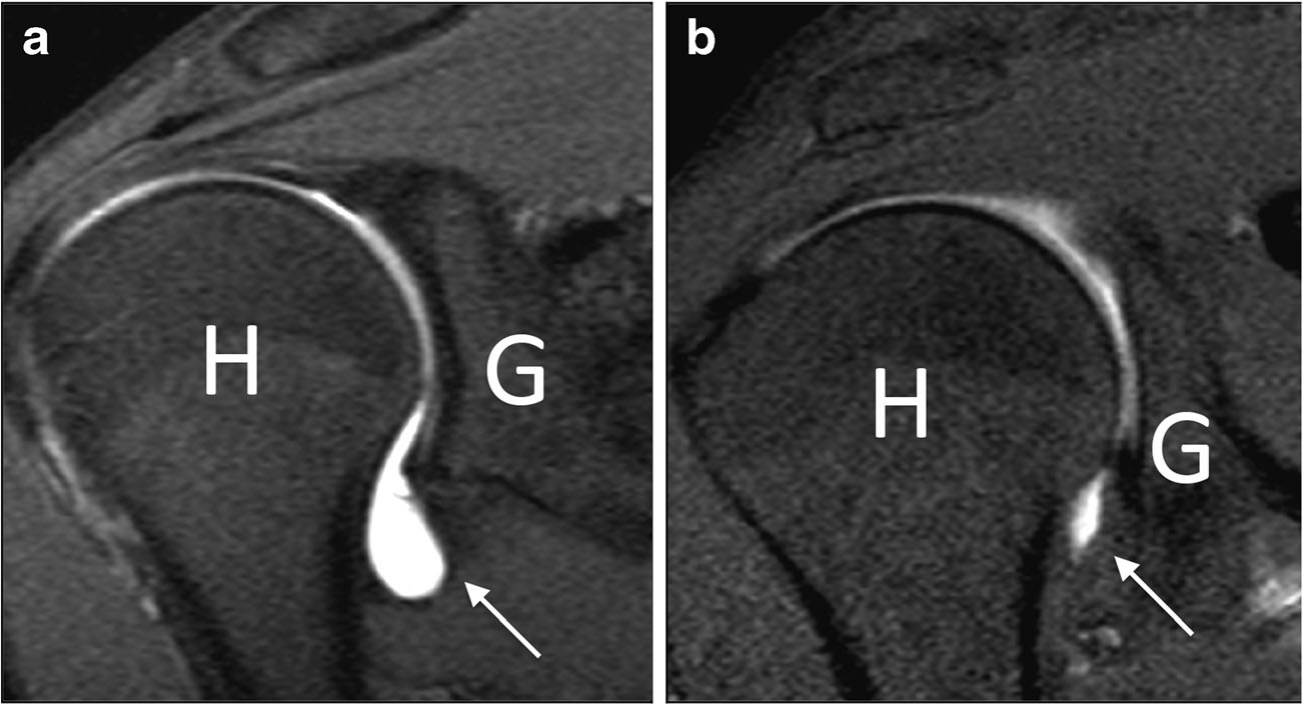
MR arthrography, coronal oblique T1-weighted fat-saturated image. (**a**) In a healthy subject, the axillary pouch is normally distensible (*arrow*). *H* humerus, *G* glenoid. (**b**). In a patient with adhesive capsulitis, the axillary pouch is contracted and poorly distended (*arrow*)

Similar to conventional arthrography, reduced axillary pouch volume on MRA suggests a diagnosis of AC (Fig. 9). Lee et al. demonstrated a mean ratio between fluid distension of the axillary and posterior recess of 0.51 in patients with AC and 0.82 in healthy controls [37]. Mengiardi et al. reported a mean axillary pouch volume of 0.52 ml in patients with AC and 0.88 ml in healthy controls [2]. Conversely, not all authors agree on the utility of evaluating capsular width at the rotator interval in patients with AC [37, 38, 42].

**Fig. 10:**
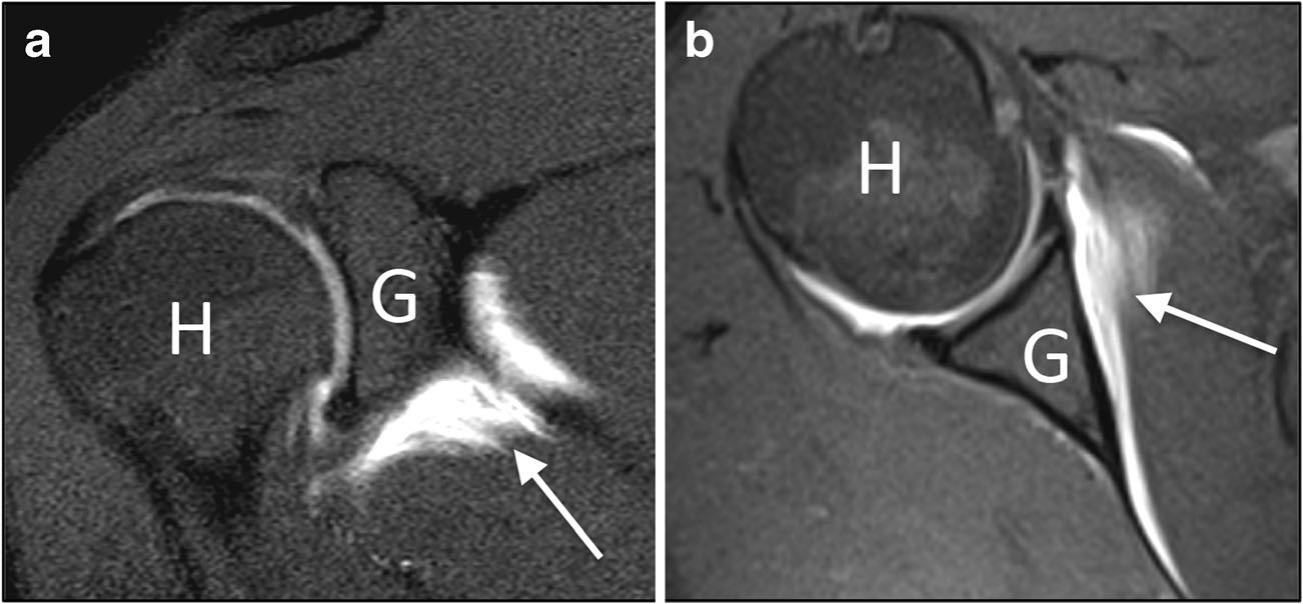
MR arthrography, (**a**) coronal oblique and (**b**) axial T1-weighted fat-saturated image in a patient with adhesive capsulitis. Leakage of contrast agent can be seen on the anterior inferior margin of the scapula (*arrow*). *H* humerus, *G* glenoid

Last, other findings reported in the literature include the leakage of contrast agent anterior to the medial margin of the scapula (Fig. 10), pseudo-synovitis over the cranial border of the subscapularis tendon and the biceps anchor, and widening of the subscapular recess [2].

Despite the volume of reported data, there is no real consensus regarding the findings that are most reliable in diagnosing AC [36]. In the authors’ experience, thickening of the coracohumeral ligament and rotator interval synovitis seen at ultrasound and the increased signal intensity of the inferior glenohumeral ligament on fat-saturated T2-weighted sequences seem to be the most conclusive.

## Conclusion

The diagnosis of AC is based mainly on clinical findings. Plain film generally plays no role in diagnosing this condition. Ultrasound can be used primarily to detect thickening of the coracohumeral ligament and synovial hypertrophy at the rotator cuff interval. MR and MRA have demonstrated high diagnostic accuracy in detecting a number of features suggestive of adhesive capsulitis, including inferior glenohumeral ligament hyperintensity, capsular and coracohumeral ligament thickening, poor capsular distension, and synovial hypertrophy and tissue scarring at the rotator interval.
